# Foodborne pathogenic bacteria in wild European hedgehogs (*Erinaceus europaeus*)

**DOI:** 10.1186/s13028-024-00747-9

**Published:** 2024-07-15

**Authors:** Maria Fredriksson-Ahomaa, Venla Johansson, Viivi Heljanko, Elina Nuotio, Heini Nihtilä, Annamari Heikinheimo, Rauni Kivistö

**Affiliations:** 1 Department of Food Hygiene and Environmental Hygiene, Faculty of Veterinary Medicine, University of Helsinki, Helsinki, Finland; 2 Korkeasaari Zoo, Helsinki, Finland; 3 Microbiology Unit, Finnish Food Authority, Seinäjoki, 60100 Finland

**Keywords:** European hedgehog, MLST, PCR, Prevalence, WGS, Zoonotic bacteria

## Abstract

**Background:**

European hedgehogs (*Erinaceus europaeus)* are widely distributed across Europe. They may play an important role by spreading zoonotic bacteria in the environment and to humans and animals. The aim of our work was to study the prevalence and characteristics of the most important foodborne bacterial pathogens in wild hedgehogs.

**Results:**

Faecal samples from 148 hospitalised wild hedgehogs originating from the Helsinki region in southern Finland were studied. Foodborne pathogens were detected in 60% of the hedgehogs by PCR. *Listeria* (26%) and STEC (26%) were the most common foodborne pathogens. *Salmonella, Yersinia*, and *Campylobacter* were detected in 18%, 16%, and 7% of hedgehogs, respectively. *Salmonella* and *Yersinia* were highly susceptible to the tested antimicrobials. *Salmonella* Enteritidis and *Listeria monocytogenes* 2a were the most common types found in hedgehogs. All *S.* Enteritidis belonged to one sequence type (ST11), forming four clusters of closely related isolates. *L. monocytogenes* was genetically more diverse than *Salmonella*, belonging to 11 STs. *C. jejuni* ST45 and ST677, *Y. pseudotuberculosis* O:1 of ST9 and ST42, and *Y. enterocolitica* O:9 of ST139 were also found.

**Conclusions:**

Our study shows that wild European hedgehogs should be considered an important source of foodborne pathogens, and appropriate hygiene measures after any contact with hedgehogs and strict biosecurity around farms are therefore important.

## Background

The European hedgehog (*Erinaceus europaeus*) is a common hedgehog species, which is widely distributed across Europe. In Finland, they live at the northernmost limit of their distribution range [1]. Hedgehogs in Finland, which apparently originate from Estonia and Russia, have been introduced by humans to new areas due to the assumption that the species kills snakes and rats [2]. European wild hedgehogs are protected by legislation in most European countries, including Finland, and keeping them as pets is therefore illegal [3]. They are nocturnal hibernators that are active during both night and day in cities. Hedgehogs are permanent residents of parks and gardens, seeking food and shelter. Householders may provide them with various refugia and supplementary food in their gardens. The European hedgehog is an insectivore that readily eats food offered by humans. Most wild hedgehogs in Finland are killed by traffic or die by starvation [4].

Zoonotic bacteria play an important role in the interactions between humans, domestic animals, and wildlife [5]. Wild hedgehogs in (sub)urban areas have been shown to carry a number of zoonotic agents and may contribute to their spread [6, 7]. Thermotolerant *Campylobacter* spp., *Salmonella enterica*, enteropathogenic *Yersinia* spp. (*Y. enterocolitica* and *Y. pseudotuberculosis*), shigatoxin-producing *Escherichia coli* (STEC), and *Listeria monocytogenes* are the most common zoonotic bacteria causing foodborne infections among humans in Europe [8]. These bacteria have sporadically been found in wild hedgehogs in Europe [3, 5, 7, 9, 10]. *Salmonella* spp. in particular has been found in both wild and pet hedgehogs [4, 11, 12]. *Salmonella* infection in hedgehogs may be a latent infection but *Salmonella* can also cause enteritis, even sudden death, especially in young animals [12]. Hedgehogs have also been linked to human salmonellosis outbreaks [11, 13, 14]. However, human infections due to wildlife contact have rarely been reported [15].

Information on the presence and characteristics of foodborne pathogenic bacteria in hedgehogs is scarce. In this study, we screened the prevalence of the most important foodborne bacteria in the faeces of (sub)urban European hedgehogs using PCR and bacterial culture. We also characterized the isolates using several methods, including whole-genome sequencing, to gain more information on the characteristics of these pathogens.

## Methods

### Samples and sample preparation

In total, 148 wild hedgehogs were studied between 2020 and 2022. The hedgehogs originated from the Helsinki region, southern Finland. They were brought to the wildlife hospital of Korkeasaari Zoo due to injuries or diseases, where they were either euthanized or died due to the severity of their conditions. Frozen carcasses of hedgehogs were transported from the zoo to the laboratory. Hedgehogs were categorized as young or adult, based on their size and body weight. Individuals with small size and body weight below 500 g were considered young.

### PCR detection of pathogenic bacteria

Real-time PCR was used to study the presence of genes carried by *Campylobacter* (*rrn*) [16], *Salmonella* (*ttr*) [17], enteropathogenic *Yersinia* (*ail*) [18, 19], STEC (*stx*1 and *stx*2) [20], and *Listeria* (*mpl*) [21]. A rectal sample of faecal matter from each hedgehog was taken with a cotton swab and mixed well with 10 ml of buffered peptone water (BPW, Oxoid, Basingstoke, UK). DNA was extracted from 1 ml of overnight enriched BPW using a Quick-DNA Fecal/Soil Microbe Miniprep kit (Nordic BioSite Oy, Helsinki, Finland). Two µl of the DNA was added to 18 µl of PCR mix consisting of 1x ready-to-use mix (iQ SYBRGreen Supermix or SsoAdvanced Universal SYBRGreen Supermix, Bio-Rad) and 200 nM of primers (metabion, Planegg, Germany). A three-step protocol (denaturation at 95 °C for 10 s, annealing at 58 °C for 10 s, and elongation at 72 °C for 30 s with 40 cycles followed by a melting curve analysis was used. The fluorescence intensity of SYBR Green was studied using the CFX96 Touch Real-Time PCR Detection System (Bio-Rad) with CFX Maestro Software v.2.3. A sample was considered positive when the threshold cycle (Ct) was below 38 and a specific melting temperature (Tm) was observed.

### Culturing of pathogenic bacteria

One hundred µl of overnight enrichment of PCR-positive *Salmonella*, *Yersinia*, and *Listeria* samples were plated on selective CHROMagar Salmonella Plus (37 °C, 24 h) (Labema, Kerava, Finland), Yersinia C.I.N Agar (30 °C, 24 h) (Oxoid), and Listeria Chromogenic Agar ALOA (37 °C, 24 h) (Labema) agar plates, respectively. *Campylobacter* presence was studied from all samples by direct plating on selective mCCDA plates (microaerophilic at 41.5 °C, 48 h) (Oxoid). Typical colonies on all selective agar plates were identified by real-time PCR using the same primers as in the PCR screening, with a threshold cycle below 30.

### Antimicrobial susceptibility in Salmonella and Yersinia isolates

Antimicrobial susceptibility of *Salmonella* and *Yersinia* isolates was tested with the Sensititre™ broth microdilution method using EUVSEC3 plates (Thermo Fisher Diagnostic, Vantaa, Finland) according to the manufacturer’s protocol. *Yersinia* isolates were incubated at 30 °C instead of 37 °C. The panel of 15 antimicrobial agents belonged to the following classes according to the European Commission’s implementing decision (EU) 2020/1729: aminoglycosides (amikacin, gentamycin), carbapenems (meropenem), cephalosporins (cefotaxime, ceftazidime), fluoroquinolones (ciprofloxacin), folate pathway antagonists (sulfamethoxazole, trimethoprim), glycylcycline (tigecycline), macrolides (azithromycin), penicillins (ampicillin), phenicols (chloramphenicol), quinolones (nalidixic acid), polymyxins (colistin), and tetracyclines (tetracycline) [22]. *E. coli* ATCC 25922 was used as a quality control microorganism. Susceptibility thresholds were interpreted in accordance with EUCAST https://www.eucast.org/fileadmin/src/media/PDFs/EUCAST_files/Breakpoint_tables/v_11.0_Breakpoint_Tables.pdf).

### Whole-genome sequencing

DNA of *Salmonella*, *Campylobacter*, *Yersinia*, and *Listeria* isolates was extracted using PureLink Genomic DNA Mini Kit (Invitrogen, Carlsbaden, CA, USA). DNA quality was measured by NanoDrop (ThermoFischer Scientific, Waltham, MA, USA), based on the 260/280 ratio, and DNA quantity by Qubit (ThermoFischer Scientific). Whole-genome sequencing (WGS) was performed for *Salmonella, Yersinia*, and *Listeria* on the Illumina DNA platform by CeGaT (Center for Genomics and Transcriptomics, Tuebingen, Germany). An Illumina DNA Prep library preparation kit and NovaSeq6000 were used to generate 100 bp paired-end reads. The short reads were assembled *de novo* using a Unicycler v.0.4.8 assembler available on the PATRIC 3.6.12 platform (https://www.bv-brc.org/app/Assembly). WGS of *Campylobacter* was performed at the Institute for Molecular Medicine Finland or Novogene (Cambridge, UK) using NoveSeqPE150. Raw sequence data were assembled using the INNUca pipeline (https://github.com/B-UMMI/INNUca).

### Characterization of Salmonella, Listeria, Yersinia, and Campylobacter isolates

Multi-locus sequence typing (MLST) based on seven housekeeping genes was performed *in silico* on the Center for Genomic Epidemiology (CGE) platform (https://cge.food.dtu.dk/services/MLST/). A more detailed comparison between 31 *Salmonella*, 29 *Listeria*, and 4 *Yersinia* isolates was performed by core genome MLST (cgMLST) targeting 3002, 1701, and 2886 genes, respectively, with Ridom SeqSphere + software v7.7.5 (Ridom GmbH, Muenster, Germany) [23]. *Salmonella* serotypes and *Listeria* serogroups were determined from the WGS data. *C. jejuni* isolates were characterized by cgMLST using chewBBACA v.3.1.2 [24]. The INNUENDO whole-genome MLST (wgMLST) schema (https://zenodo.org/record/1322564#.YHVvO-gzY2w), consisting of 2794 loci, was downloaded from chewie-NS [25]. Minimum spanning trees (MST), representing pairwise allele distances, were used to visualize allelic differences (Ads) between the isolates. *Salmonella* and *Listeria* isolates forming a cluster (CL) displayed a maximum of 10 ADs from each other. The CLs were shaded in grey, and the number of ADs between the isolates was indicated on the connecting line.

### Statistical analyses

The analyses were performed with IBM SPSS Statistics 29.0.1 (IBM, Armonk, NY, USA). All tested variables were categorical. Fisher’s exact test was used to compare the differences between the proportions of two groups.

## Results

A total of 148 wild European hedgehogs were studied, 64 of which (43.2%) were young and 84 (56.8%) were adults (Table 1). All hedgehogs originated from the Helsinki region, most (70.9%, 105/148) from Helsinki City and the rest (29.1%, 43/148) from the surrounding areas, with a maximum radius of 65 km from Helsinki City. These hedgehogs died or were euthanized in 2020 (39.2%, 58/148), 2021 (37.8%, 56/148), or 2022 (23.0%, 34/148). Most (63.5%, 94/148) of the hedgehogs were injured, and the rest (36.5%, 54/148) were sick, including exhausted and emaciated animals.

**Table 1:** Number of hedgehogs from the Helsinki region (southern Finland) studied between 2020 and 2022

Sampling year	All	Age		Sampling area (Helsinki region)		
		Young	Adult	Helsinki City	Surroundings^a^	
2020	58 (39.2%)	16 (27.6%)	42 (72.4%)	44 (75.9%)	14 (24.1%)	
2021	56 (37.8%)	35 (62.5%)	21 (37.5%)	37 (66.1%)	18 (33.9%)	
2022	34 (23.0%)	13 (38.2%)	21 (61.8%)	24 (70.6%)	10 (29.4%)	
2020-22	148	64 (43.2%)	84 (56.8%)	105 (70.9%)	43 (29.1%)	

^a^A maximum radius of 65 km from Helsinki City

Foodborne pathogens were frequently detected in the faeces of 148 hedgehogs from the Helsinki region (Table 2). In total, 89 (60.1%) of the hedgehogs were positive for at least one foodborne pathogen species in their faeces. *Listeria* (26.4%) and STEC (25.7%) were the most common pathogens detected in the faeces by PCR. *Salmonella* and *Campylobacte*r were detected in 17.6% and 6.8% of the hedgehogs, respectively. Enteropathogenic (*ail*-positive) *Yersinia* was detected in 16.2% of the hedgehogs, of which *Y. pseudotuberculosis* and *Y. enterocolitica* were recovered in 20 (13.5%) and 4 (2.7%) faecal samples, respectively.

**Table 2:** Detection of foodborne pathogens in 148 wild hedgehogs in southern Finland by PCR

Pathogen	Gene	Positive hedgehogs		
		No.	%	95% CI
*Campylobacter*	*rrn*	10	6.8	3.3–12.1
*Salmonella*	*ttr*	26	17.6	11.8–24.7
*Yersinia*	*ail*	24	16.2	10.7–23.2
STEC	*stx*	38	25.7	18.9–33.5
*Listeria*	*mpl*	39	26.4	19.5–34.2
At least one pathogen		89	60.1	51.8–68.1

The prevalence of foodborne pathogens in the faecal samples varied between young animals and adults (Table 3). *Salmonella* was detected more often (*P* = 0.05) in young animals (25.0%) than in adults (11.9%), while STEC was more commonly (*P* = 0.02) found in adults (33.3%) than in young animals (15.6%). In 2022, *ail*-positive *Yersinia* was detected significantly (*P* = 0.04) more often in young hedgehogs (61.5%) than in adults (23.8%). No significant differences were observed between the pathogen prevalences in injured and sick hedgehogs. The prevalences of most pathogens differed clearly between the sampling years (Table 3). Only *Listeria* was quite evenly distributed during the years; its prevalence varied between 25.0% and 29.4%. *Salmonella* was most (26.8%) frequently detected in 2021, while *Yersinia* (38.2%) and STEC (47.1%) were frequent findings in 2022. Significant differences were observed between the sampling years in the prevalence of *Yersinia* and STEC (Table 3).

**Table 3:** Distribution of foodborne pathogens among 148 wild hedgehogs depending on the grouping

Groups		Campylobacter		Salmonella		Yersinia		STEC		Listeria	
		*N*	%	*N*	%	*N*	%	*N*	%	*N*	%
Age	Young	2	3.1	16^a^	25.0	11	17.2	10^a^	15.6	21	32.8
	Adult	8	9.5	10^b^	11.9	13	15.5	28^b^	33.3	18	21.4
Health status	Injured	5	5.3	16	17.0	16	17.0	24	25.5	22	23.4
	Sick	5	9.3	10	18.5	8	14.8	14	25.9	17	31.5
Sampling area	Helsinki City	9	8.6	19	18.1	19	18.1	26	24.8	30	28.6
	Surroundings	1	2,3	7	16.3	5	11.6	12	27.9	9	20.9
Year	2020	1	1.7	7	12.1	2^a^	3.4	9^a^	15.5	15	25.9
	2021	7	12.5	15	26.8	9^a, b^	16.1	13^a, b^	23.2	14	25.0
	2022	2	5.9	4	11.8	13^b^	38.2	16^b^	47.1	10	29.4

Superscripts ^a^ and ^b^ differed significantly (*P* ≤ 0.05) from each other

*Campylobacter* was isolated from four hedgehogs: *C. jejuni* from three and *C. lari* from one hedgehog (Table 4). Using MLST based on seven housekeeping genes, two sequence types (ST45 and ST677) were identified among *C. jejuni* isolates, and the *C. lari* isolate belonged to ST21. *Salmonella* was isolated from all PCR-positive samples. In total, 31 *Salmonella* isolates were identified from 26 (17.6%) hedgehogs. Most isolates (93.5%, 29/31) were identified as serotype Enteritidis and two (6.5%) as Typhimurium (Table 4). All *S.* Enteritidis isolates belonged to ST11 and both Typhimurium isolates to ST19. All *Salmonella* isolates were susceptible to all 15 tested antimicrobial agents. *Yersinia* was isolated from four (16.7%, 4/24) PCR-positive hedgehogs. One animal was *Y. enterocolitica* serotype O:9 positive and three were *Y. pseudotuberculosis* O:1 positive in their faeces. Two STs (ST9 and ST42) were identified among *Y. pseudotuberculosis* O:1 isolates, and the *Y. enterocolitica* O:9 isolate belonged to ST139. All *ail*-positive *Yersinia* isolates were susceptible to most (14/15) tested agents: *Y. enterocolitica* was only resistant to ampicillin and *Y. pseudotuberculosis* to colistin. *L. monocytogenes* was isolated from 29 (19.6%) hedgehogs and from most (74.4%, 29/39) PCR-positive samples. Most isolates (79.3%, 23/29) belonged to serogroup 2a. Serogroup 4b was identified in six (20.7%) hedgehogs. *L. monocytogenes* 2a isolates belonged to eight STs (ST7, 18, 37, 177, 398, 451, 585, and 2488) and *L. monocytogenes* 4b isolates belonged to three STs (ST1, 4, and 382). The most common STs of serogroups 2a and 4b were ST2488, found in nine hedgehogs, and ST1, found in five hedgehogs, respectively. PCR-positive STEC samples were not studied further by culturing.

**Table 4:** Multi-locus sequence types (MLSTs) identified among foodborne pathogenic bacteria isolated from hedgehogs between 2020 and 2022 in Helsinki region, southern Finland

Isolation year	No. of isolates	Species	Serotype	MLST^a^
2020 (*n* = 1)	1	*Campylobacter jejuni*		677
2021 (*n* = 3)	2	*Campylobacter jejuni*		45
	1	*Campylobacter lari*		21
2020 (*n* = 7)	6	*Salmonella*	Enteritidis	11
	1		Typhimurium	19
2021 (*n* = 19)	18	*Salmonella*	Enteritidis	11
	1		Typhimurium	19
2022 (*n* = 5)	5	*Salmonella*	Enteritidis	11
2022 (*n* = 4)	1	*Yersinia pseudotuberculosis*	O:1b	9
	2		O:1a	42
	1	*Yersinia enterocolitica*	O:9	139
2020 (*n* = 13)	3	*Listeria monocytogenes*	4b^a^	1
	1		4b	4
	1		2a	7
	1		2a	18
	2		2a	37
	1		4b	382
	4		2a	2488
2021 (*n* = 9)	2	*Listeria monocytogenes*	2a	398
	1		2a	451
	2		2a	585
	4		2a	2488
2022 (*n* = 7)	1	*Listeria monocytogenes*	4b	1
	1		1/2a	37
	1		1/2a	177
	2		1/2a	398
	1		1/2a	585
	1		1/2a	2488

^a^Serogroup

**Fig. 1:**
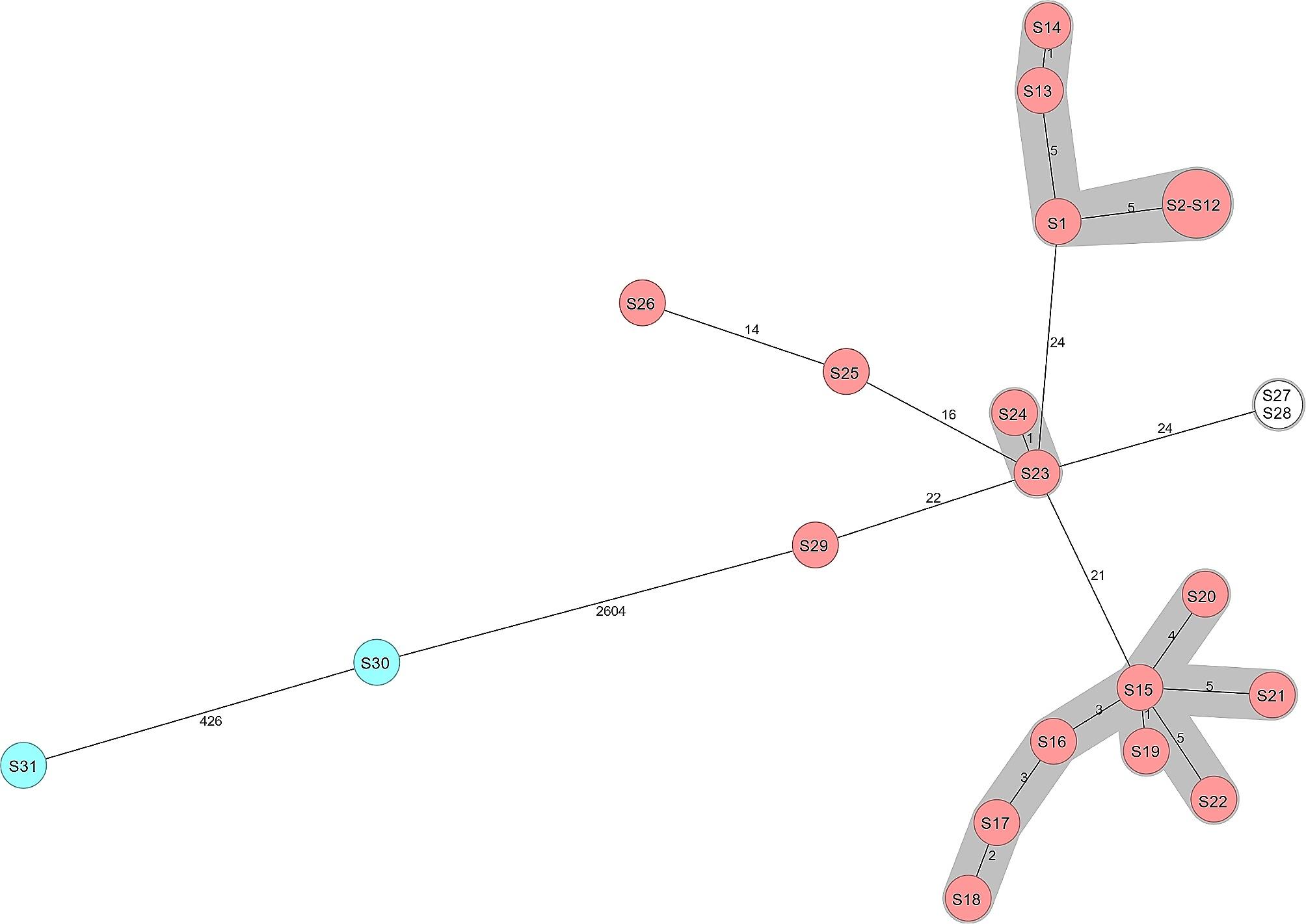
Minimum spanning tree of 31 *Salmonella* isolates from wild hedgehogs in southern Finland during 2020-22. Nodes are numbered according to Table 5. Number of allelic differences (ADs) between the isolates are indicated on the connecting lines. Clusters are shaded in grey and a cluster distance threshold of maximum 10 ADs was used

In total, 29 *S.* Enteritidis and 2 *S.* Typhimurium isolates were characterized with Ridom cgMLST. Most (99.7%, 26/29) *S.* Enteritidis isolates formed four clusters, with 0 to 5 ADs in each cluster (Fig. 1). Most isolates (11/14) in the largest cluster were highly related, with no ADs, including 14 *S.* Enteritidis isolates (S1–S14). These isolates were found from hedgehogs sampled during different years and from different areas in the Helsinki region (Table 5). The two *S.* Typhimurium isolates differed from each other with 426 allelic differences.

**Table 5:** *Salmonella enterica* and *Listeria monocytogenes* isolates characterized with cgMLST

Isolate	ST^a^	Serotype	AD^b^	Year	Helsinki region
*Salmonella enterica*					
S1–S14	11	Enteritidis	0–6	2020–21	Helsinki City, Surrounding^c^
S15–S22	11	Enteritidis	1–8	2020–22	Helsinki City
S23, S24	11	Enteritidis	1	2022	Helsinki City
S25	11	Enteritidis	16	2021	Helsinki City
S26	11	Enteritidis	14	2021	Helsinki City
S27, S28^d^	11*	Enteritidis	0	2021	Surrounding
S29	11	Enteritidis	22	2020	Surrounding
S30, S31	19	Typhimurium	426	2020–21	Helsinki City, Surrounding
*Listeria monocytogenes*					
L1–L4	1	4b^e^	20–76	2020, 2022	Helsinki City
L5	382	4b	1029	2020	Helsinki City
L6	4	4b	1064	2020	Helsinki City
L7–L10	398	2a	9–25	2021–22	Helsinki City
L11–L13	585	2a	12–13	2021–22	Helsinki, Surrounding
L14–L16	37	2a	13–28	2020–21	Helsinki City, Surrounding
L17	177	2a	1170	2022	Surrounding
L18	18	2a	1201	2020	Helsinki City
L19	451	2a	1247	2021	Helsinki City
L20	7	2a	1257	2020	Helsinki City
L21–L29	2488	2a	0–8	2020–21	Helsinki City, Surrounding

^a^Sequence type based on seven housekeeping genes

^b^Allelic differences

^c^A maximum radius of 65 km from Helsinki City

^d^From the same animal

^e^Serogroup

**Fig. 2:**
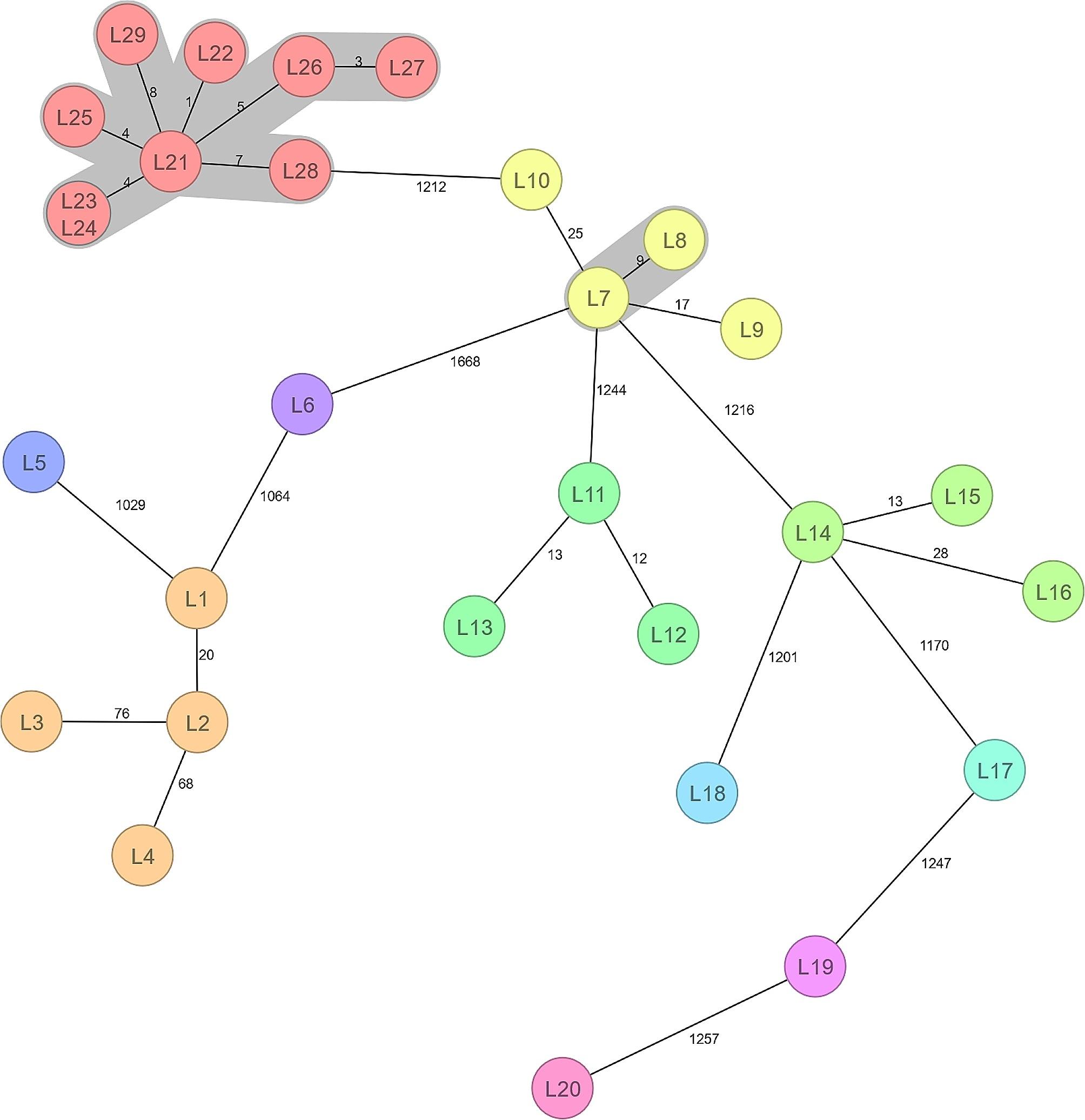
Minimum spanning tree of 29 *Listeria monocytogenes* isolates from wild hedgehogs in southern Finland during 2020-22. Nodes are numbered according to Table 5. Number of allelic differences (ADs) between the isolates are indicated on the connecting lines. Clusters are shaded in grey and a cluster distance threshold of maximum 10 ADs was used

In total, 29 *L. monocytogenes* isolates were analysed with Ridom MLST. Eleven (37.9%) *L. monocytogenes* 1/2a isolates formed two clusters with 1 to 9 ADs (Fig. 2). The largest cluster, with 1 to 8 ADs, was formed by *L. monocytogenes* isolates (L21–L29) belonging to the sequence type ST2488. These isolates originated from hedgehogs sampled during different years and from different areas (Table 5). Only two isolates (L7 and L8) with 9 ADs belonged to ST398 in the second cluster. These two isolates originated from hedgehogs sampled in 2020 and 2021 from Helsinki City. The two *Y. pseudotuberculosis* O:1a isolates belonging to ST42 showed 29 allelic differences with Ridom MLST analysis. The only *Y. pseudotuberculosis* O:1b (ST9) isolate differed from the nearest O:1a (ST42) isolate with 1129 allelic mismatches. INNUENDO cgMLST analysis revealed 334 ADs (among 1070 shared loci) between the two *C. jejuni* ST45 isolates, indicating that the two strains were clearly distinct and not closely related to each other.

## Discussion

Our study shows that wild European hedgehogs are an important source of foodborne zoonotic bacteria. We detected the zoonotic bacteria by PCR, which is a very sensitive method, detecting small numbers of bacteria [26]. Approximately 60% of the hedgehogs excreted at least one pathogenic bacterial species in their faeces. The faeces samples were collected from carcasses of sick or injured hedgehogs, which may have affected the high prevalence. The most frequently detected species were *L. monocytogenes* and STEC. Both pathogens have rarely been reported in hedgehogs before; however, only a few studies have been conducted previously [5, 9]. We isolated *L. monocytogenes* from 20% of the faecal samples. In an earlier study, this pathogen was found in extra-intestinal sites from 2% of the tested hedgehogs [9]. We did not find any significant temporal or regional differences in *Listeria* prevalence. We also found no significant difference between young and adult animals. Our study demonstrates that *L. monocytogenes* is commonly excreted in the faeces of hedgehogs. *L. monocytogenes* is ubiquitous in the environment [27], which indicates that hedgehogs can contract this pathogen from the environment through eating soil-dwelling invertebrates. The high PCR prevalence of STEC in the hedgehogs was interesting; however, this topic requires further study, as we did not have any isolates for characterization, which is needed for estimating isolate pathogenicity. STEC has not been reported in hedgehogs before.

*Salmonella* was detected in the faecal samples of 26 (18%) hedgehogs by PCR, and it was also isolated from all these samples, indicating that the number of *Salmonella* in the faeces was quite high. A prevalence of 18% is high in countries like Finland, where *Salmonella* prevalence is very low (< 1%) in meat-producing animals (pigs, ruminants, and poultry) [28]. One reason for the high prevalence in the wild hedgehogs may be that our samples originated from injured and sick animals. However, the high prevalence was not a surprise because *Salmonella* has been isolated from 57% of dead hedgehogs in Finland in an earlier study [4]. Reasons behind the very high prevalence in this earlier study are probably due to the very restricted sampling area and because liver samples were used. Salmonellosis in hedgehogs is assumed to mostly be a latent infection found in the liver [12]. *Salmonella* prevalence in the faeces of European hedgehogs in Europe has been low (3–4%) in earlier studies [5, 29]. One reason for the high *Salmonella* prevalence in the hedgehog faeces in our study may be that the animals originated from (sub)urban areas. Urban hedgehogs have been suggested to carry more zoonotic pathogens than those in rural areas [30]. All the hedgehogs in our study had been injured or sick, which is a stress factor that may have activated the latent infection and increased the excretion of *Salmonella* in their faeces. *Salmonella* was also significantly more often detected in young hedgehogs than in adults.

Our study showed that wild European hedgehogs are also possible carriers of *Campylobacter* and pathogenic (*ail*-positive) *Yersinia.* However, both pathogens were quite rarely isolated from the faecal samples. Only a few earlier studies [7, 10, 31] have reported *C. jejuni* and *Y. pseudotuberculosis* in hedgehogs. Using PCR, we detected *Y. pseudotuberculosis* (14%) more frequently than *Y. enterocolitica* (3%) in the faecal samples. Wild animals are assumed to be the most important reservoir for *Y. pseudotuberculosis* [32]. Isolation of *Yersinia*, especially, *Y. pseudotuberculosis* is very challenging, which may be one reason for the low isolation rate.

We only found two *Salmonella* serotypes in hedgehog faeces: Enteritidis and Typhimurium. These serotypes are the most prevalent serotypes responsible for human salmonellosis in the EU [8], and they are also the most frequent serotypes reported in hedgehogs [3, 7, 13, 29]. In Denmark, *S.* Enteritidis isolates found in hedgehogs were shown to belong to the same clonal lineage as isolates found in infected humans [33]. Enteritidis was frequently found in wild hedgehogs in our study. It was the only serotype found in the hedgehogs in Finland in a previous study [4]. All Enteritidis isolates found in our study belonged to ST11. This type was reported to be common in hedgehogs and humans in the UK [29]. In Germany, *S.* Enteritidis ST183 was associated with hedgehogs [15]. Most (90%) of our *S.* Enteritidis ST11 isolates formed four clusters with closely related isolates. Interestingly, most (79%) isolates in the largest cluster were highly related, with no ADs, even if they differed temporally and geographically from each other. This indicates that we have endemic *S*. Enteritidis isolates circulating among hedgehogs in the Helsinki region, despite the hedgehogs not necessarily being epidemiologically linked to each other. In two hedgehogs, we also identified Typhimurium, which belonged to ST19 and clearly differed (426 ADs) from each other. Hedgehogs are suggested to be a reservoir for *S.* Typhimurium, and they have also been linked to two human salmonellosis outbreaks caused by Typhimurium in Norway [13]. Outbreaks due to S. Typhimurium have also been linked to pet hedgehogs [11, 14, 34]. We could not show any antimicrobial resistance among *Salmonella* isolates in our study. Also, no evidence of antimicrobial resistance was present in the hedgehog Enteritidis isolates in the UK [29].

*L. monocytogenes* isolates recovered in our study belonged to serogroups 2a and 4b, which are commonly found in animals and humans. These types have also been identified in wild animals in Finland [35] and from the livers of wild hedgehogs in the UK [9]. We found several STs among serotype 2a and 4b isolates, especially among 2a isolates, which dominated in the hedgehogs in our study. Most of the STs identified in this study have been found in wild animals in our earlier study, indicating that these STs (ST1, ST4, ST7, ST18, ST37, ST451, and ST585) circulate in wildlife in southern Finland [35]. Interestingly, several isolates belonging to ST2488 formed a cluster with only 1 to 8 ADs. These isolates mostly differed from each other both temporally and regionally. This sequence type could be an endemic type adapted to hedgehogs in the Helsinki region.

We identified three *C. jejuni* isolates, which belonged to ST45 and ST677. Both types have been predominant among domestically acquired human infections [36, 37] and in broilers in Finland [38]. In addition, ST45 has been found from various other sources, including cattle, sheep, pigs, cats and dogs, wild birds, and environmental waters worldwide (PubMLST *Campylobacter jejuni/coli* isolate database) [39]. On the other hand, ST677 has been linked to more severe bacteraemia cases, in addition to gastroenteritis, in Finland [40]. Although mainly found in humans, ST677 has also been found, albeit infrequently, from other sources, with wild birds and chickens likely being the main reservoirs. We also found *C. lari* ST21 from one hedgehog. Overall *C. lari* is an uncommon finding in human infections compared to *C. jejuni* and *C. coli* [8]. However, ST21 has been identified as the most prevalent genotype among human *C. lari* infections in Europe [41] and the USA [42]. It has also been found from various sources (animals, food, and environment among others) [41]. Overall, wild birds, especially seagulls, and shellfish are considered to be the main reservoirs of *C. lari* [43]. Although clinically relevant *Campylobacter* spp. and genotypes were identified among hedgehogs in our study, they were relatively infrequent findings. Furthermore, only a few samples were culture positive, suggesting that hedgehogs are unlikely to play a major role in the epidemiology of *Campylobacter* species in humans.

*Y. pseudotuberculosis* isolates found in three hedgehogs were identified as ST9 (O:1b) and ST42 (O:1a), and they were not closely related to each other based on cgMLST. However, both STs are common types in wildlife and have also been found in wild and domestic animals in Finland [44, 45]. Additionally, one pathogenic (*ail*-positive) *Y. enterocolitica* was found and it was identified as O:9, which is the second most common serotype found in humans [8]. This serotype is associated with ruminants and has been found in sheep and voles in Finland [46, 47]. The *ail*-positive *Yersinia* isolates were susceptible to almost all the antimicrobial agents tested in our study. The *Y. enterocolitica* O:9 isolate was only resistant to ampicillin. *Y. enterocolitica* is naturally resistant to this agent due to β-lactamase production. *Y. pseudotuberculosis* O:1 isolates were resistant only to colistin. Colistin resistance has been shown to be common in *Y. pseudotuberculosis* due to mutations in genes responsible for their lipopolysaccharide structure [48].

Wild European hedgehogs, especially stressed or sick ones, may contribute to the spread and transmission of important foodborne pathogens due to the high prevalence in their faeces. As hedgehogs often live in (sub)urban areas, people handling and feeding hedgehogs may expose themselves to foodborne pathogens carried by hedgehogs. A high prevalence of *Salmonella*, especially among hedgehogs sampled at feeding places, has been reported in Norway [13]. The same study suggested that hedgehog feeding places may serve as sites of *Salmonella* transmission between animals. Appropriate hygienic measures after any contact with hedgehogs are recommended to reduce the risk of enteric pathogen transmission from hedgehogs to humans [34]. Hedgehogs inhabiting parks and gardens with close contact to humans and other animals may pose a public health hazard that needs to be investigated further.

## Conclusions

Our results suggest that wild European hedgehogs should be considered an important source of foodborne zoonotic bacteria. *S.* Enteritidis ST11 was isolated from the faeces of several injured and sick wild hedgehogs originating from various parts in the Helsinki region, indicating that this pathogen is endemic and widely distributed in European hedgehogs in southern Finland. *Salmonella* monitoring in hedgehogs around farms, especially in countries with low *Salmonella* prevalence in farm animals, could be one way of obtaining information on the *Salmonella* situation around farms. Strict farm biosecurity is crucial to prevent *Salmonella* and other foodborne pathogens from entering the food production chain.

## Data Availability

The data analysed during the study are available from the corresponding author on reasonable request.

## Declarations

### Prior publication

Data have not been published previously.
